# Association between Obesity and History of Abuse among American Indians in Rural California

**DOI:** 10.4172/2165-7904.1000208

**Published:** 2014-02-10

**Authors:** Felicia Hodge, M Susan Stemmler, Karabi Nandy

**Affiliations:** Department of Health Policy and Management, University of California, USA

**Keywords:** Obesity, American indians, Body Mass Index (BMI), Childhood trauma

## Abstract

**Objectives:**

To explore factors associated with obesity among American Indians.

**Methods:**

A cross-sectional survey of American Indian adults (N=459) was conducted at 13 rural reservation sites in California. Participants responded to a survey about their health and wellness perceptions. The Body Mass Index (BMI) was used to assess obesity. A predictive model for BMI was built using a generalized regression model.

**Results:**

Having high blood pressure and having a history of verbal abuse in childhood were significant predictors of higher BMI. Participants with high blood pressure were likely to have 3.2 units of BMI higher on average than those who do not have high blood pressure (p-value <0.0001). Similarly, those with a history of childhood verbal abuse were likely to have 1.9 units higher BMI on average compared to those with no such history. Having a history of diabetes or sexual abuse in childhood trend towards increased BMI, although not statistically significant.

**Conclusion:**

Identifying childhood trauma and its impact on adult obesity rates among American Indians provides new avenues for intervention. Efforts to reduce over weight and obesity should include culturally sensitive interventions to ameliorate and repair what is lost through personal violations of stigma, abuse or neglect.

## Introduction

Obesity is a significant problem in the United States; 35.7% of adults in the country meet the criteria for obesity [1]. Prevention of obesity has become a national public health priority for all ethnic and age groups. Although preventive efforts to mitigate obesity have somewhat stabilized its prevalence [2], obesity stubbornly proliferates within certain vulnerable population groups [3] and contributes to additional risks for health disparities [4,5]. American Indians have been living with an obesity epidemic for the last three generations such that prevalence rates within certain tribal groups far exceed that of the general population [6,7]. National health profiles document American Indian and Alaska Native adults as 1.6 times more likely than White adults to be obese [6,8]. Obesity among American Indians has escalated to a high of 40–60% within certain tribes [9]. As a result, tribal groups have been the focus of studies to determine factors that contribute to their excess weight gain [9,10]. Research has focused on the effects of cultural transitions from traditional ways of life to modern day lifestyles [11] and has led to insights about disparities among American Indians as identified through historical traumas, geographical isolation, and lack of resources. These factors characterize some of the socioeconomic stressors and scarcity for many American Indians living on tribal lands despite the relative wealth of the general population [9].

A transition away from traditional ways of life has been repeatedly referenced as one of the key contributors to obesity among American Indians [12–14]. Traditional life typified by rugged daily activities and eating patterns dependent on seasonal and natural resources have given way to more sedentary day to day activities and the ready availability of processed foods and government commodities. Referencing this transition, epigenetic theories have postulated futures of childhood and adult obesity for American Indians with complications of type 2 diabetes and cardiovascular disease [10,15].

Researchers have approached the public health problem of obesity with programs that propose education coupled with diet and exercise activities [14,15]. Various interventional trials promoting behavior change have been conducted in American Indian communities with the intent of preventing and mitigating health consequences associated with obesity. However, health consequences related to obesity, such as type 2 diabetes, cardiovascular disease, dyslipidemia, joint and mobility problems and early death continue unabated. The interventional trials report moderate successes in their attempts to modify food selection, dietary intake volume, and exercise among the targeted Indian tribal groups [13,16]. Only a limited number of health researchers have conducted nutritional assessments of modern day American Indians through the lens of cultural traditions and with the cooperation of tribal leaders [17,18]. Despite targeted health messages and interventional trials, predictions for behavioral modification and successful weight management among American Indians remain guarded [13].

Recent studies, not including American Indians participants, have reported an association between obesity and a history of trauma or adverse events in childhood [19,20]. These research studies have assessed individual categories of events, particularly physical and verbal abuse, neglect, and sexual abuse as adverse events in childhood. This paper examines correlates of obesity among American Indians considering the effects of trauma brought about by history of neglect or abuses.

## Methods

Institutional Review Board approvals were obtained from the University of California at Berkeley, San Francisco and Los Angeles, the University of Minnesota, and the national Indian Health Service. Tribal approvals were obtained either by letter or by resolution of support.

### Sample and setting

A cross-sectional survey was conducted in 13 reservation sites in rural California settings. Prior to data collection, we had estimated that a sample size of 500 from all clinics combined would power our study at 90%. Based on clinic utilization data, the number of subjects from each clinic averaged 50. Five hundred American Indian adults were recruited from a sampling frame constructed from Indian outpatient clinic registries. Recruitment for the study was coordinated through the clinic registries. A random sampling of households yielded 459 adult residents each representing a household. Participant inclusion criteria required the clinic registry to specify American Indian ethnicity, age 18 years or older, whether resident of the local reservation, and whether clinic user within the past 5 years. Study participants consented via written forms. Participants were carefully informed that their participation in the study was voluntary and that they did not have to answer any question that they did not want to answer. In addition, they were told that clinic services did not depend upon their participation in the study. Lastly, candidates were informed that they could decide to withdraw from the study at any time if they chose to do so. To preserve confidentiality of the participants, personal identifiers were encoded to blind the research team from linking survey responses to specific individuals.

The survey took approximately 60 minutes to complete and each respondent received a non-monetary incentive for their participation. Surveys were completed in the respondent’s home or in a private room at the local clinic.

The survey was organized into seven sections: demographic characteristics and American Indian cultural constructs, health status and wellness, health problems, obesity, preventive and risky behaviors, mental health and adverse events.

### Demographic characteristics

Questions posed to characterize the sample included gender, age, employment status, education and marital status.

### Health and wellness status

A single question was used to measure general health status: “How would you rate your health, nowadays? Would you say that it is excellent, very good, fair or poor?” A single question also measured wellness perception: “How would you rate your wellness? Would you say that it is excellent, very good, fair or poor?” Both measures were dichotomized into two categories (good = excellent/very good and poor = fair/poor).

### Health problems

Respondents were asked to check off any personal ailments from a list of common health problems ranging from cancer and cardiovascular disease to type2 diabetes, physical disabilities and mental health problems. They were also encouraged to add to the list any health problem, if their own health disorder was not included in the list.

### Obesity status

Obesity measures were based on the body mass index (BMI), which is calculated from the reported height and weight of the respondent. Underweight is defined as a BMI score of less that 18.5; healthy weight is a BMI between 18.5 to 24.9; overweight is a BMI between 25 to 29.9; obese is a BMI score of 30–39.9 [21] and morbidly obese is a BMI score above 40 [22].

### Preventive and risky behaviors

Respondents marked from a list of behaviors that included: exercise, diet, smoking cigarettes, alcohol drinking, drug usage, practicing safe sex and driving wearing a seatbelt.

### Adverse events

A series of questions asked respondents if they had ever experienced adverse events in the form of physical abuse, sexual abuse, verbal abuse or neglect, and they were asked to specify the age at which the trauma occurred. The designated ages were later categorized as during childhood, adolescence or adulthood.

### Data analysis

The outcome variable of interest was respondents’ body mass index (BMI). BMI’s lower than 19 or greater than 50 were considered outliers and were left out of the analyses. A predictive model for BMI was built using a generalized regression model. It was based on a potential set of predictors including demographic characteristics, health indicators, preventive and/or risky behaviors, cultural constructs and adverse life events such as history of abuse and/or neglect throughout lifetime. The covariates were entered in an initial full model and a final model was obtained through iterative elimination, a process of entering meaningful variables and eliminating redundant ones from an initial model. Covariates included variables that were associated with the outcome at the .15 level in preliminary analyses; covariates were retained if they were significant at the .10 level. Certain variables were retained in the final regression models even if they were not statistically significant because they were considered important variables for control for in modeling obesity. Model fit was assessed using R-squared and adjusted R-squared statistics. All model assumptions were checked and met. All statistical analyses were performed with a Statistical Analysis Program (SAS/STAT). Statistical significance was set at p<0.05 (2-tailed).

## Results

### Overall sample

Socio-demographic characteristics for the overall sample are shown in Table 1. The median age of American Indians in the overall sample was 42 years (inter-quartile range of 24), 71% were females and 54% were married. Thirty-three percent spoke their tribal language, 87% were enrolled in a tribe, and 43% reported that they had 50% or more Indian blood quantum. Employment status was reported by 62% of the sample. Of the American Indian respondents in the overall sample who responded to the obesity questions, the average BMI was 31 (range: 19.1–49.1), which falls in the obese category. One fifth (n=96) of the respondents had completed less than a high school education.

### Predictors of BMI

Having high blood pressure and a history of verbal abuse in childhood are the two significant predictors of higher BMI. In particular, those who have high blood pressure are likely to have 3.2 units of BMI higher on average than those who do not have high blood pressure (t_1_=4.05, p – value <0.0001), all other factors remaining constant. Similarly, those with a history of childhood verbal abuse are likely to have 1.9 units higher BMI on average compared to those who do not have such history (t_1_=2.22, p – value =0.03), all other factors remaining the same. Having a history of diabetes or sexual abuse in childhood indicate increased BMI, although they were not statistically significant (Table 2). This model controlled for depression.

## Discussion

Findings from this study of American Indians in rural California settings offer more insights into factors associated with the high prevalence of obesity in this population. We found that higher BMI in our sample was associated with a health history of high blood pressure (p=0.0001) and adverse events (specifically, verbal abuse in childhood, p=0.03). In addition, we found that sexual abuse in childhood (p=0.09) may also be associated with increased BMI, although it was not statistically significant. Other recent studies have also found an association between obesity and adverse events, particularly physical and sexual abuse [23,24]. Traumas, including neglect and verbal abuse, reported in all phases of the lifespan, should be studied in the context of obesity.

Having a history of diabetes (17.87%) was also found to trend (p=0.08) towards having an increased BMI. The kinds of conditions that predispose individuals to eating disorders and obesity include poor eating habits and sedentary lifestyles, both of which may be mediated to reduce BMI rates. Most research about obesity among American Indians has concentrated on the harmful effects of the modern day diet of high fat, sugar-laden, process foods and sedentary lifestyle related to secondary health consequences like diabetes and cardiovascular disease [15,25]. While deviations away from traditional food selection and preparation, sedentary lifestyle, and lack of availability of nutritious, unprocessed foods in American Indian communities may account for some of the behavioral aspects of obesity, these factors alone do not address the broader context of life as an American Indian or the personal traumas that may lead to coping achieved through eating and inactivity.

Multifactorial aspects of obesity have been uncovered, including genetic influences, and environmental influences that stimulate intrauterine fetal programming for obesity [26,27]. We would be remiss if we did not acknowledge these previous findings, but we must also state that further research is still needed [16,28,29]. Associated health problems such as cardiovascular disease (CVD) and stroke that would also benefit from better understanding of the predictors for obesity. Screenings such as waist-hip ratio and fasting glucose measures could potentially identify a cadre of obesity indicators that also contribute to diabetes, CVD, stroke and other metabolic diseases. For American Indians, the risks of these problems are associated with high BMI rates and they demand prevention and early detection through screenings.

The results of this study align with recent research that explores psychological and social correlates to better understand the less obvious factors and behaviors that influence obesity. The impact of adverse events on obesity need to be evaluated more closely [30]. Posing sensitive questions about adverse events needs to be a regular practice when making assessment regarding maladaptive eating patterns and physical disorders associated with obesity. As important as diet counseling and physical fitness interventions are for obesity, culturally sensitive counseling regarding adverse events must to be considered in the American Indian community.

Identifying the types of childhood trauma and its influence on obesity later in adulthood among American Indians provide new avenues for understanding and developing possible interventions. Efforts to intervene to reduce overweight and obesity should be directed toward prevention and through correction of conditions that predispose to disordered eating and obesity. Further research is needed to verify our findings and for developing practice-related research to evaluate and establish culturally relevant interventions for the American Indian people. In the health care and social service settings, it becomes very important to maintain sensitive awareness surrounding disclosure of personal trauma and violence. Culturally sensitive efforts to ameliorate and repair what is lost through stigma, abuse or neglect are also needed when they accompany obesity. Parental and community/tribal guidance to prevent abuse and neglect of children and women are called for within the American Indian communities.

## Figures and Tables

**Table 1:** Characteristics of the overall sample of adult American Indians (N=385).

Sample Characteristics (n=459)		
		% (n)
**Demographics**		
Age	Median (Interquartile range, n)	42.72 (23.6, 426)
Gender	Male (n) Female (n)	25.8% (117) 74.2% (336)
Marital Status	Married/Living with Divorced/Separated/ Single	52.4% (232) 47.6% (211)
Employment	Yes No	62.5% (275) 37.5% (165)
Education	Less than high school High school or more	21.7% (96) 78.3% (347)
**Health**		
Body Mass Index	Mean (Standard error, n)	31.02 (0.33, 385)
Health Perception	Fair/ Poor Very good/excellent	59.2% (267) 40.8% (184)
Wellness Perception	Positive Negative	72.6% (321) 27.4% (121)
Hx of Diabetes	Yes N0	17.8% (79) 82.1% (363)
Hx of Hypertension	Yes No	32.2% (141) 67.8% (297)
**Health Behaviors**		
Smoking Status	Current Former Never	39.3% (170) 26.3% (114) 34.4% (149)
Intent to Quit Smoking (Current)	Yes No	26.5% (40) 73.5% (111)
Alcohol Problem	Yes No	36.2% (162) 63.7% (284)
Drug Problem	Yes No	11.7% (50) 88.3% (378)
Uses Safer Sex Practices	Yes No	45.7% (134) 54.3% (159)
Monogamous Relationship	Yes No	87.7% (270) 12.3% (38)
Hx Suicidal Ideation	Yes No	20.4% (89) 79.6% (347)
Attempted Suicide	Yes No	7.9% (35) 92.2% (411)
**Cultural/Social Life**		
Satisfied with spiritual life	Yes No	88.6% (381) 11.4% (49)
Speaks tribal language	Yes No	32.6% (136) 67.4% (281)
Speaks tribal language at home	Yes No	26.4% (121) 73.6% (338)
Participates in AI Practices	Yes No	58.4% (260) 41.6% (185)
Practices specific to tribe	Most Some None	41.7% (161) 43.0% (166) 15.2% (59)
Participated in healing ceremony	Yes No	34.2% (149) 65.8% (287)
Active in AI community	Yes No	62.3% (269) 37.8% (163)
Feel connected to AI community	Yes No	83.5% (329) 16.5% (65)

**Table 2:** Parameter Estimates from Regression Model for BMI.

Variable	df	Parameter Estimate	Standard Error	t-statistic	P-value
Constant	1	33.32	2.18	15.31	<0.0001
Blood Pressure	1	3.27	0.81	4.05	<0.0001
Verbal Abuse in childhood	1	1.85	0.83	2.22	0.03
Sexual abuse in childhood	1	1.77	1.04	1.71	0.09
Diabetes	1	−1.68	0.97	−1.74	0.08
Depression Rarely Sometimes Occasionally	1 1 1	−0.76740 −0.40701 −2.63159	1.71535 1.88880 2.06621	−0.45 −0.22 −1.27	0.6549 0.8295 0.2037
